# Unified framework for brain connectivity-based biomarkers in neurodegenerative disorders

**DOI:** 10.3389/fnins.2022.975299

**Published:** 2022-09-20

**Authors:** Sung-Woo Kim, Yeong-Hun Song, Hee Jin Kim, Young Noh, Sang Won Seo, Duk L. Na, Joon-Kyung Seong

**Affiliations:** ^1^Department of Bio-Convergence Engineering, Korea University, Seoul, South Korea; ^2^Department of Artificial Intelligence, Korea University, Seoul, South Korea; ^3^Department of Neurology, Samsung Medical Center, School of Medicine, Sungkyunkwan University, Seoul, South Korea; ^4^Department of Health Sciences and Technology, Samsung Advanced Institute for Health Sciences & Technology (SAIHST), Sungkyunkwan University, Seoul, South Korea; ^5^Department of Digital Health, Samsung Advanced Institute for Health Sciences & Technology (SAIHST), Sungkyunkwan University, Seoul, South Korea; ^6^Department of Neurology, Gil Medical Center, Gachon University of College of Medicine, Incheon, South Korea; ^7^Neuroscience Research Institute, Gachon University, Incheon, South Korea; ^8^Department of Intelligent Precision Healthcare Convergence, Sungkyunkwan University, Seoul, South Korea; ^9^Alzheimer's Disease Convergence Research Center, Samsung Medical Center, Seoul, South Korea; ^10^Neuroscience Center, Samsung Medical Center, Seoul, South Korea; ^11^School of Biomedical Engineering, Korea University, Seoul, South Korea; ^12^Interdisciplinary Program in Precision Public Health, Korea University, Seoul, South Korea

**Keywords:** brain connectivity, connectivity-based biomarker, biomarker scores, connected component, Laplacian regularization, Kendall's rank correlation, Alzheimer's disease

## Abstract

**Background:**

Brain connectivity is useful for deciphering complex brain dynamics controlling interregional communication. Identifying specific brain phenomena based on brain connectivity and quantifying their levels can help explain or diagnose neurodegenerative disorders.

**Objective:**

This study aimed to establish a unified framework to identify brain connectivity-based biomarkers associated with disease progression and summarize them into a single numerical value, with consideration for connectivity-specific structural attributes.

**Methods:**

This study established a framework that unifies the processes of identifying a brain connectivity-based biomarker and mapping its abnormality level into a single numerical value, called a biomarker abnormality summarized from the identified connectivity (BASIC) score. A connectivity-based biomarker was extracted in the form of a connected component associated with disease progression. BASIC scores were constructed to maximize Kendall's rank correlation with the disease, considering the spatial autocorrelation between adjacent edges. Using functional connectivity networks, we validated the BASIC scores in various scenarios.

**Results:**

Our proposed framework was successfully applied to construct connectivity-based biomarker scores associated with disease progression, characterized by two, three, and five stages of Alzheimer's disease, and reflected the continuity of brain alterations as the diseases advanced. The BASIC scores were not only sensitive to disease progression, but also specific to the trajectory of a particular disease. Moreover, this framework can be utilized when disease stages are measured on continuous scales, resulting in a notable prediction performance when applied to the prediction of the disease.

**Conclusion:**

Our unified framework provides a method to identify brain connectivity-based biomarkers and continuity-reflecting BASIC scores that are sensitive and specific to disease progression.

## 1. Introduction

The brain is composed of structurally and functionally intertwined neurons. This pattern of interconnected neurons can be disrupted or reinforced by the progression of neurodegenerative disorders (Zhan et al., 2016; Lee et al., 2021b; Schumacher et al., 2021; Caravaglios et al., 2022). The development of various neuroimaging devices, such as magnetic resonance imaging (MRI), electroencephalography, and near-infrared spectroscopy, has allowed for non-invasive extraction of the brain's connectivity. The introduction of graph-theoretical methods has allowed for quantitative analysis of the complex structure of the brain's connectivity. Consequently, altered brain connectivity can be quantified, leading to the discovery of connectivity-based biomarkers.

Measurability, particularly the ability to be measured on a continuous scale, is a critical characteristic of diagnostic biomarkers, given their common objectives of explanation and prediction (Shmueli, 2010; Bang, 2020). This need is even more pronounced in neurodegenerative disorders, where multiple pathological phenomena interact in a complex manner (Gomez-Ramirez and Wu, 2014; Jack et al., 2018), complicating attempts to make a diagnosis using a single biomarker. Thus, constructing a single abnormality score for a biomarker can be effectively integrated with other clinical manifestations, such as elucidating the mechanism of the disorder through structural equation modeling or predicting whether a person has the disease through logistic modeling. Furthermore, expressing biomarker abnormalities as a single numerical value assists physicians and patients in intuitively comprehending patients' disease states.

Predictive models, such as linear or logistic regression models, can be used to define biomarker abnormalities, which can be summarized as model responses indicating the level of abnormality (Shen et al., 2017; Lee et al., 2018, 2021a). These models aim to map multiple variables constituting the biomarker to a target variable, either the presence/absence of diseases or a proxy measure representing disease stages. Accurately predicting the target variable in model development may restrict the association between disease stage and response. For example, minimizing the mean squared error (MSE) in linear predictive models maximizes the association but is limited to a linear relationship. If a disease is represented by a dichotomous variable, the summarized score can be constructed by minimizing the binary cross-entropy (BCE). Nevertheless, this score should ideally be either zero or one, which is incompatible with the fact that the abnormality level reflects continuously changing disease stages. It is also challenging to construct a single model response associated with disease progression when the disease is classified as having three or more consecutive stages.

The structural attributes inherent in biomarkers should also be considered when dealing with them. One attribute of neuroimaging biomarkers is derived from the fact that brain changes can occur over a continuous domain, causing interconnected brain changes in contiguous regions (Lindquist, 2008). A spatially positive correlation has been accounted for by representing disease-related alterations as cluster-like structures comprising adjacent voxels (Poline et al., 1997; Worsley, 2001). This concept has been extended from physical to topological cluster in connectivity-based analysis, where the alteration is mathematically modeled as a graph, and the cluster-like structure is defined as a connected component composed of multiple adjacent edges (Zalesky et al., 2010, 2012; Han et al., 2013). However, spatially autocorrelation should also be considered between the levels of alteration of the edges that comprise the connected component.

This study aimed to establish a framework that unifies the processes of identifying brain connectivity-based biomarkers and mapping abnormality levels into a single numerical value, termed a biomarker abnormality summarized from the identified connectivity (BASIC) score. The framework consists of two successive steps: extraction of altered brain connectivity related to neurodegenerative disorders, and construction of a scoring function with respect to connectivity-based biomarkers. In contrast to predictive models employing MSE or BCE as the objective for model construction, this scoring function was constructed by maximizing Kendall's rank correlation coefficient (Kendall, 1938) between its value and the disease stage, which can be represented by continuous, or even discrete variables having ordinal scales. Especially when the disease stage is represented as a discrete variable, Kendall's correlation is invariant on the nominal scale for data with binary or ordinal scale; therefore, the association with the disease stage can be measured so long as the nominal scale is maintained. Neighborhood information in brain connectivity is considered in the form of connected components that explicitly reflect spatially positive correlations. We constructed the BASIC scores associated with the progression of Alzheimer's disease (AD) as an example of neurodegenerative disorder, and validated that the scores are sensitive and specific to the disease trajectory. We also validated that the scores also reflect continuous disease-related alterations, resulting in noticeable prediction performance when used to predict the disease.

## 2. Materials and methods

### 2.1. Dataset information

Two independent datasets were utilized in this study. Participants in Datasets 1 and 2 underwent MRI scans and neuropsychological tests at Samsung Medical Center and Gachon University Gil Medical Center, respectively. Table 1 details the demographic and clinical information for each dataset.

**Table 1:** Demographic information according to the stage of Alzheimer's disease continuum.

	**Dataset 1**					**Dataset 2**	
	**CU**	**EMCI**	**LMCI**	**MildAD**	**ModAD**	**CU**	**AD**
*N*	116	57	141	173	39	63	92
Age, years^*^	65.86 (8.63)	69.63 (7.86)	70.58 (8.86)	71.18 (9.08)	72.51 (9.59)	66.02 (10.97)	68.26 (10.75)
Sex (female), no.^*†*^	58	32	82	115	30	29	65
Education level, years^*^	12.07 (4.88)	10.36 (6.41)	11.22 (4.79)	8.95 (5.54)	8.63 (5.66)	12.29 (4.49)	8.57 (4.69)
ICV,ℓ^*^	1.40 (0.22)	1.37 (0.29)	1.34 (0.22)	1.38 (0.21)	1.39 (0.21)	1.50 (0.15)	1.46 (0.16)
MMSE^*^	28.73 (1.39)	26.95 (2.66)	25.69 (2.81)	20.40 (4.16)	13.74 (5.16)	28.02 (2.02)	17.11 (6.41)
CDR-SOB^*^	0.51 (0.39)	1.10 (0.79)	1.40 (0.98)	4.34 (1.88)	10.68 (3.69)	0.00 (0.00)	5.23 (3.23)
Aβ deposition							
Global SUVR^*^	N/A					0.41 (0.11)	1.00 (0.22)
Positivity (Aβ+), no.^*†*^	N/A					2	88

CU, Cognitively unimpaired; EMCI, Early mild cognitive impairment; LMCI, Late mild cognitive impairment; AD, Alzheimer's disease; MildAD, Mild AD; ModAD, Moderate-to-severe AD; ICV, Intracranial volume; MMSE, Mini-Mental State Examination; CDR-SOB, Clinical Dementia Rating scale-Sum Of Boxes; SUVR, Standardized uptake value ratio.

* Data are given as the mean with standard deviation in parentheses.

† Data are given as the number of category in parentheses.

Dataset 1 included 116 cognitively unimpaired (CU) individuals, 198 patients with amnestic mild cognitively impairment (MCI), and 212 patients with AD. Patients with MCI and AD were further divided into two subgroups: 57 patients with early MCI (EMCI) and 141 patients with late MCI (LMCI) for the MCI group; and 173 patients with mild AD (MildAD) and 39 patients with moderate-to-severe AD (ModAD) for the AD group. These subgroups were categorized based on clinical criteria, such as diagnostic criteria for MCI/AD and neuropsychological tests. See the previous study for more information on participant recruitment and subgroup division (Kim et al., 2021). Dataset 2 consisted of 63 CU individuals and 92 AD patients. Similarly, AD patients in Dataset 2 were identified according to clinical diagnostic criteria, which were described in the previous study in conjunction with the recruitment and inclusion/exclusion criteria (Lee et al., 2021b).

CU participants were further divided into two subgroups based on their ages: young CU (YC) if their age at recruitment was greater than or equal to 65, and old CU (OC) otherwise. Patients with MCI and AD were also subdivided based on onset age: early-onset MCI (EOMCI) or early-onset AD (EOAD) if symptoms developed before age 65, and late-onset MCI (LOMCI) or late-onset MCI (LOAD) otherwise. According to these criteria, individuals in Dataset 1 were classified into 46 YC, 69 EOMCI, 75 EOAD, 70 OC, 129 LOMCI, and 137 LOAD; individuals in Dataset 2 were classified into 33 YC, 51 EOAD, 32 OC, and 41 LOAD.

### 2.2. MR image acquisition

For Dataset 1, both T1-weighted MRI and resting-state functional MRI (rsfMRI) data were acquired using a 3.0-Tesla Intera Achieva MRI scanner (Philips Medical Systems, Best, the Netherlands) as previously described (Kim et al., 2016, 2021).

For Dataset 2, the T1-weighted MRI data were acquired using a 3.0-Tesla MRI scanner (Verio, Siemens, Erlangen, Germany) as previously described (Lee et al., 2021b). The rsfMRI data was acquired for 9 min using gradient-echo echo planar imaging (EPI) with the following parameters: TR = 3,000 ms, TE=30 ms, and voxel size = 3.4 × 3.4 × 3.4 mm^3^. During the acquisition of rsfMRI data, participants were instructed to keep their eyes open, not move their heads, and refrain from falling asleep.

### 2.3. Image preprocessing

The T1-weighted MRI data were preprocessed according to the standard pipeline in FreeSurfer v5.1 to obtain intracranial volume (ICV). The rsfMRI data were preprocessed using FEAT (FMRI Expert Analysis Tool) and FAST (FMRIB's Automated Segmentation Tool) in FSL (FMIRB's Software Library) v5.0 (Jenkinson et al., 2012) as described in the previous study (Kim et al., 2021).

### 2.4. PET image acquisition and quantification

Participants in Dataset 2 also underwent [^18^F]-Flutemetamol positron emission tomography (PET) scans with a Siemens Biograph 6 Truepoint PET/computed tomography (CT) scanner (Siemens, Knoxville, Tennessee, United States) using a list-mode emission acquisition. We computed the global amyloid standardized uptake value ratio (SUVR) from each PET image by averaging AD-related regions, including the prefrontal, superior/inferior parietal, lateral temporal, and anterior/posterior cingulate cortices with pons as the reference region. Amyloid positivity was defined as the global SUVR > 0.62. Detailed information on PET imaging parameters and SUVR computation are demonstrated in the previous study (Lee et al., 2021b).

### 2.5. Functional connectivity network construction

A whole-brain functional connectivity network (FCN) for the *i*-th participant was mathematically modeled as a finite, simple, undirected, and vertex-labeled graph $G_{i}=\left(V,E_{i}\right)$, where V is a set of vertices and $E_{i}\in V\times V$ is a set of edges. V was defined as the volumetric regions of interest (ROIs) determined using the Automated Anatomical Labeling (AAL) brain atlas (Tzourio-Mazoyer et al., 2002). The ROIs consist of 40 cerebral cortical regions and five subcortical regions for each hemisphere, resulting in |V|=90. The presence of an edge $e\in E_{i}$, with a weight of *c*_*e*_, representing its connection strength, was determined by temporal associations between regional blood oxygenation level dependent signals, which were computed by averaging the signals of the voxels included in each ROI. Pearson product-moment correlation coefficient (Pearson's *r*) was used for the association measure, followed by the Fisher *r*-to-*z* transformation. A negative coefficient was set to zero, as used in our previous studies (Kim et al., 2017, 2021), because of its ambiguous biological interpretation (Fox et al., 2009; Murphy et al., 2009; Rubinov and Sporns, 2010; Cao et al., 2014). Though, our framework described below is not limited to this thresholding criterion. For convenience, we considered the nonexistent edge $e\notin E_{i}$ to be equivalent to the edge $e\in E_{i}$ with *c*_*e*_ = 0.

### 2.6. A unified framework for connectivity-based biomarkers

#### 2.6.1. Overview

**Figure 1:**
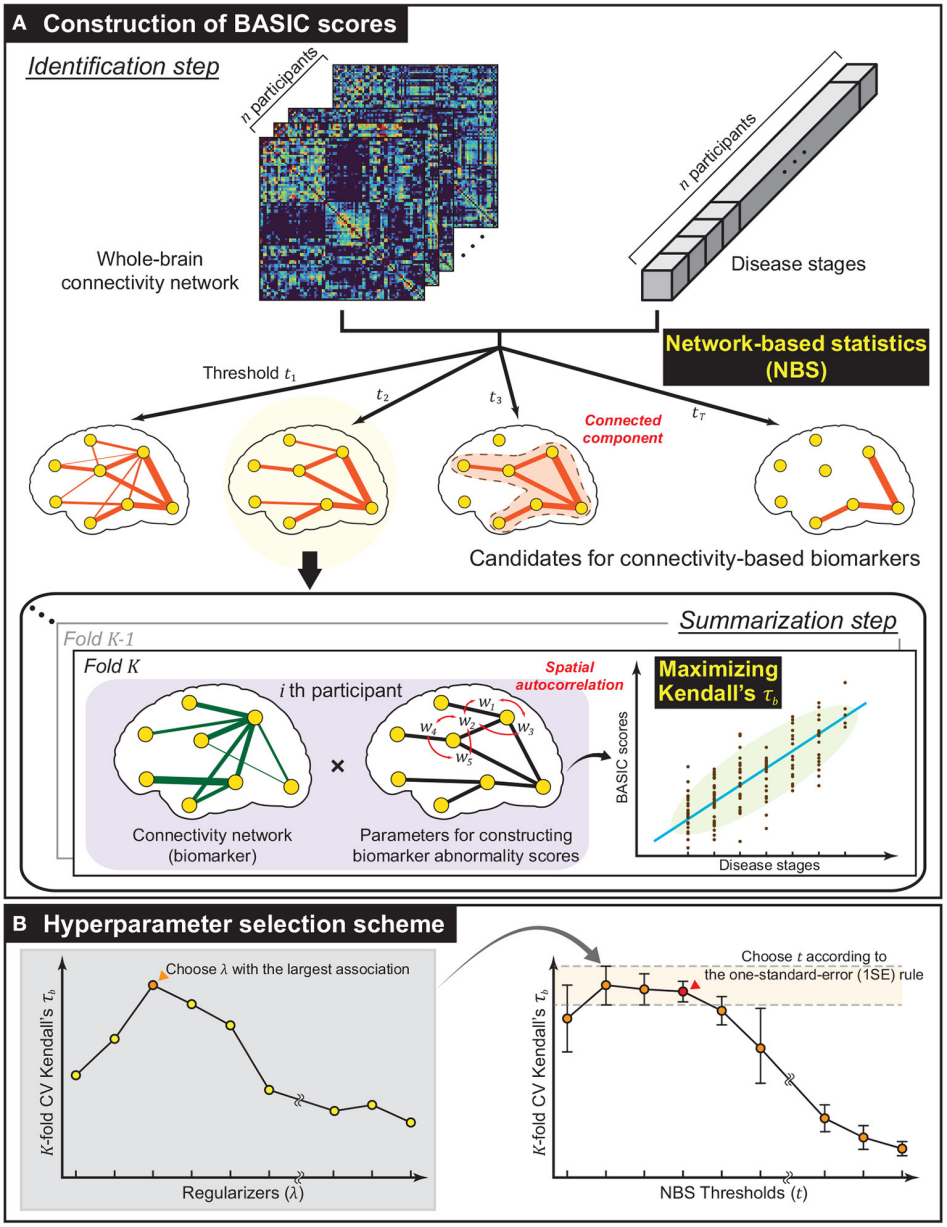
Methodological overview. **(A)** The framework established for constructing biomarker abnormality scores associated with disease progression based on brain connectivity. Using network-based statistics (NBS), a collection of biomarker candidates was extracted in the form of connected components of varying extent in the identification step. Next, in the summarization step, Biomarker Abnormality Summarized from the Identified Connectivity (BASIC) scores were constructed in a manner that maximizes Kendall's rank correlation (τ_*b*_) to disease stages. In this step, the spatially positive correlation was considered as a regularization in optimization. **(B)** To improve generalized performance, the biomarker and parameters for constructing BASIC scores were determined based on cross-validation (CV). The optimal regularizer that yielded the best CV Kendall's τ_*b*_ for all predefined NBS thresholds was chosen. The optimal NBS threshold was then selected according to the one-standard-error (1SE) rule.

Let $D={\left\{\left(G_{i},d_{i}\right)\right\}}_{i=1}^{n}$ be a dataset consisting of the whole-brain connectivity network $G_{i}=\left(V,E_{i}\right)$, where *d*_*i*_ is the realization of a reference variable *D* representing the stage of a disease measured on continuous, dichotomous, or ordinal scales. A *connectivity-based biomarker*, denoted as H=(S,F), was defined as a connected component of an unweighted graph $\bar{G}=\left(V,E_{1}\cup \cdots \cup E_{n}\right)$. The abnormality score of H was defined as a continuous and bounded real number. More formally, we defined a *scoring function* s:G→[0,1], which produces a *biomarker abnormality score* from $G_{i}\left[F\right]=\left(V_{i}^{\prime },E_{i}^{\prime }\right)$, an edge-induced subgraph of $G_{i}$ consisting of $E_{i}^{\prime }\subset E_{i}$ with vertices incident to $e\in E_{i}^{\prime }$. The framework was designed to identify H based on D, and obtain each individual's biomarker abnormality score $s\left(G_{i}\left[F\right]\right)$, the BASIC score, by finding the optimal *s* according to two consecutive steps: *identification* and *summarization*. This process is schematically illustrated in Figure 1A.

#### 2.6.2. Identification step

This step extracts a set of possible connectivity-based biomarkers based on D. To satisfy the condition that H is connected, we employ a network-based statistic (NBS) that identifies the statistically significant connected component for specific hypothesis testing (Zalesky et al., 2010). In the NBS, statistics for the testing, such as *t*-statistics, correlation coefficients, and regression coefficients, were calculated on all edges $e\in E_{1}\cup \cdots \cup E_{n}$. Edges whose statistics exceeded a predefined threshold *t* were chosen, and the connected components comprising suprathreshold edges were extracted. This procedure was repeated *m* times, with the sample being generated from randomization of the original one, resulting in different statistics for all edges; thus, it yielded distinct connected components with maximum sizes. It gives a null distribution of the maximum extent of connected components, and statistical significance was determined at the level of connected components rather than at the individual edges.

Edge-level statistics were computed using a general linear model. For each edge $e\in E_{1}\cup \cdots \cup E_{n}$, *c*_*e*_ was used as a dependent variable, and the reference variable *D*, together with an intercept and nuisance variables, were used as independent variables. If *D* is measured on a continuous scale, then the variable of interest has a single continuous value; otherwise, the variables of interest are made up of dummy variables. Dummy variables were encoded using a staircase coding scheme, particularly when *D* was measured on an ordinal scale. To generate randomly permuted samples, we employed the Freedman-Lane procedure (Freedman and Lane, 1983; Winkler et al., 2014), where the permutation was performed on the residual of the reduced model containing only nuisance variables.

#### 2.6.3. Summarization step

H is summarized as a single numerical value in this step. In constructing *s*, the following conditions were considered: a) if a sequence of the scores $\left\{s\left(G_{i}\left[F\right]\right)\right\}$ was associated with that of the corresponding disease stage {*d*_*i*_}, b) if the connectivity-specific structural attribute, a spatially positive correlation on adjacent edges, was explicitly reflected, and c) if inf*s* = 0, and sup*s* = 1. Complying with these conditions, we defined *s* = Φ_μ, σ_ ◦ *f*, where *f* is a real-valued function on G, called a *summarizing function*, and Φ_μ, σ_ is a cumulative distribution function (CDF) of a normal distribution with mean μ and standard deviation σ, which are computed as the sample mean and sample standard deviation of ${\left\{s\left(G_{i}\left[F\right]\right)\right\}}_{i=1}^{n}$, respectively.

In this study, we defined *f*(·;**w**), parameterized by a column vector $\text{w}\in {\mathbb{R}}^{\left|F\right|}$, as the linear combination of the edge connection strengths, that is,


(1) $$\begin{array}{l} f\left(G_{i}\left[F\right];\text{w}\right)=\underset{e\in E_{i}^{\prime }}{\sum }w_{e}\cdot {\left(c_{e}\right)}_{i}={\text{w}}^{T}{\text{c}}_{i}, \end{array}$$

where *w*_*e*_ is a parameter of *f* for an edge *e*, and $\text{c}\in {\mathbb{R}}^{\left|F\right|}$ is a column vector whose element is *c*_*e*_ for an edge *e*. Notably, Equation (1) can be applied to graphs with different sets of edges because we set *c*_*e*_ = 0 for even $e\in F\backslash E_{i}$. **w** was computed by maximizing the association between the values of *f* and the corresponding ones for *D*, considering the connectivity-specific spatial autocorrelation simultaneously, or formally satisfying the following objective:


(2) $$\begin{array}{l} \underset{w}{\text{minimize}}\;-R\left(w^{T}c_{i},d_{i};D\right)+\lambda ∥Bw{∥}_{2}^{2}, \end{array}$$

where R(·,·;D) is the measure of association between two univariate variables in the dataset D, $\text{B}\in {\left\{-1,0,1\right\}}^{\left|S|\times |F\right|}$ is an oriented vertex-edge incidence matrix, and λ is a Tikhonov regularizer. For R, Kendall's tau-b (τ_*b*_) was used (Kendall, 1938). In contrast to Pearson's *r*, τ_*b*_ can be used for the association between two variables measured on a dichotomous or ordinal scale, as well as for identifying nonlinear monotonic relationships beyond linear relationships (Chok, 2010). Further, as a rank statistic, τ_*b*_ is more resistant to outliers when calculated for continuous variables (Couso et al., 2018). The second term is for regularization, which can be rewritten as follows:


(3) $$\begin{array}{l} {\text{w}}^{T}{\text{B}}^{T}\text{B}\text{w}=\underset{u,v\in F:u∽v}{\sum }{\left(w_{u}-w_{v}\right)}^{2}, \end{array}$$

where *u* ∽ *v* means that the edges *u* and *v* are adjacent. Note that the **B**^*T*^**B** is equal to the edge-based graph Laplacian, and the regularization term results in Laplacian regularization (Ando and Zhang, 2006). In other words, spatial autocorrelation is added in the form of minimizing of the difference between the parameter values of adjacent edges.

We solved the optimization problem with the objective (Equation 2) using the GRID algorithm that was known to find a solution comparable to the globally optimal one (Croux et al., 2007; Alfons et al., 2017). It deals with a slightly modified version of objective (Equation 2), which has an additional equality constraint ∥**w**∥_2_ = 1, resulting in a new optimization problem to find the optimal direction of **w**. Finally, the final objective is stated as follows:


(4) $$\begin{array}{l} \begin{array}{c} \underset{\text{w}}{\text{minimize}}\;-R\left({\text{w}}^{T}c_{i},d_{i};D\right)+\lambda ∥\text{B}\text{w}{∥}_{2}^{2},\\ \text{subject to }∥\text{w}{∥}_{2}=1. \end{array} \end{array}$$

In the first iteration of this algorithm, the optimal direction was determined by performing a grid search that divides the angular intervals [−π/2, π/2) into *n*_*g*_ equal parts alternately for each coordinate. At *r*th iteration, the search interval was reduced to [−π/2^*r*^, π/2^*r*^).

Finally, the BASIC score for a given whole-brain connectivity network $G_{i}$ is calculated as ${\hat{\text{w}}}^{T}{\text{c}}_{i}$, where $\hat{\text{w}}$ is the optimal solution of the optimization problem (Equation 4).

#### 2.6.4. Hyperparameter selection scheme

The BASIC score aims to improve generalized performance in association with disease progression by selecting the value of hyperparameters based on cross-validation (CV). Two hyperparameters were selected for the proposed framework: *t* and λ. The former controls the extent of the connectivity-based biomarker, analogous to controlling the sparsity of parameters of *f*. The latter controls for the degree of spatial positive correlation. The optimal values of the hyperparameters were determined by simultaneously varying their values within predefined sets.

For a fixed value of *t*, *K*-fold CV was performed at each value using a set of predefined values of λ, and the value with the highest averaged τ_*b*_ across *K* folds was selected. Then, *t* was chosen such that the corresponding subnetwork exhibits the most parsimonious and comparable average τ_*b*_ according to the one-standard-error (1SE) rule (Hastie et al., 2009). The scheme for selecting the values of *t* and λ is illustrated in Figure 1B.

### 2.7. Experiments

To validate whether the framework performs well when *D* is measured on a continuous or ordinal scale, we identified connectivity-based biomarkers related to various stages of AD and Aβ deposition from whole-brain FCNs using the optimal values of *t* and λ determined by *K*-fold CV. We also computed the corresponding BASIC scores using the entire dataset. Table 2 provides detailed information on the experimental settings. The optimal value of *t* was determined by computing the generalized association performance of sub-networks containing 0.1–10% of the total number of possible edges (i.e., from four to 401) for values within its range because the NBS is ineffective and has a low statistical power at too high or too low threshold (Zalesky et al., 2010).

**Table 2:** List of experimental settings.

**Experiment**	**1**	**2**	**3**	**4**	**5**
Disease/Pathology	AD	AD	AD	EOAD/LOAD	Aβ deposition
Reference variable (*D*)	Dichotomous	Ordinal	Ordinal	Ordinal	Continuous
Subgroups	CU, AD	CU, MCI, AD	CU, EMCI, LMCI, MildAD, ModAD	YC, YOMCI, YOAD / OC, LOMCI, LOAD	
Dataset (D)	Dataset 1	Dataset 1	Dataset 1	Dataset 1	Dataset 2
Hyperparameters					
**Identification step**					
Edge-level statistics	partial *F*-statistics				
No. of permutation (*m*)	10,000				
NBS threshold (*t*)	${\left\{k\right\}}_{k=1}^{30}$	${\left\{0.5k\right\}}_{k=1}^{30}$	${\left\{0.5k\right\}}_{k=1}^{40}$	${\left\{k\right\}}_{k=1}^{30}$	${\left\{k\right\}}_{k=1}^{20}$
Nuisance variables	age, sex, education level, and ICV				
**Summarization step**					
No. of iterations (*n*_*r*_)	100				
No. of grid points (*n*_*g*_)	20				
No. of folds (*K*)	5				
Regularizer (λ)	{*e*^−15^, *e*^−14^, …, *e*^1^, *e*^0^}				

CU, Cognitively unimpaired; MCI, Mild cognitive impairment; EMCI, Early MCI; LMCI, Late MCI; AD, Alzheimer's disease; MildAD, Mild AD; ModAD, Moderate-to-severe AD; NBS, Network-based statistics; ICV, Intracranial volume.

When *D* was measured on a dichotomous scale, we compared the BASIC scores with scores determined by the logistic model, where the score function was defined as *s* = *g* ◦ *f* with a sigmoid function *g*(*x*) = 1/(1 + *e*^−*x*^). In contrast to the BASIC score, where **w** satisfies the objective with respect to the *summarizing* function *f*, the logistic model-based scores were obtained by minimizing the BCE between $s\left(G_{i}\left[F\right]\right)=g\left(f\left(G_{i}\left[F\right];\text{w}\right)\right)$ and *d*_*i*_ ∈ {0, 1} as the target variable. For *D* with continuous values, we compared the BASIC scores with scores determined by optimizing the least-square form of the classical canonical correlation with Laplacian regularization (Sun et al., 2008).

#### 2.7.1. Statistical analysis

Continuous biomarker abnormality scores in two successive groups along a disease continuum were compared using the Student's *t*-test and Welch's *t*-test. The false discovery rate (FDR) procedure was used for each experiment to correct for multiple comparisons (Benjamini and Hochberg, 1995). To examine the relationship between biomarker abnormalities and cognitive function, we calculated the coefficients of multiple correlations between the biomarker score and clinical scores, including the mini-mental state examination (MMSE) and the clinical dementia rating scale-sum of boxes (CDR-SOB) scores. Statistical significance of the coefficient of multiple correlations was determined by permutation testing with 10,000 permutations.

#### 2.7.2. Classification analysis

Support vector machine with radial basis function kernel and uniform class prior probability was used for binary classification. It is run by MATLAB R2021b (www.mathworks.com), with all other hyperparameters set to default.

## 3. Results

The optimal values of the hyperparameters and corresponding results for all experiments are presented in Table 3.

**Table 3:** Selected hyperparameter values and the corresponding results for each experiment.

	**Diseases**	**Disease stages**	**Objective functions**	**Regularizations**	***t***	**λ**	**No. of edges**	5-fold CV performances^*^
Exp. 1	AD	2 stages	Kendall's τ_*b*_	Laplacian	9	*e* ^−10^	215	0.5493 (0.0308)^*†*^
Exp. 2	AD	3 stages	Kendall's τ_*b*_	Laplacian	6	*e* ^−4^	160	0.4899 (0.0549)^*†*^
Exp. 3	AD	5 stages	Kendall's τ_*b*_	Laplacian	4	*e* ^−10^	132	0.3993 (0.0305)^*†*^
Exp. 4a	EOAD	3 stages	Kendall's τ_*b*_	Laplacian	8	*e* ^−12^	80	0.5427 (0.0889)^*†*^
Exp. 4b	LOAD	3 stages	Kendall's τ_*b*_	Laplacian	7	*e* ^−12^	39	0.4192 (0.0523)^*†*^
Exp. 5	Aβ	Continuous	Kendall's τ_*b*_	Laplacian	8	*e* ^9^	126	0.4839 (0.0566)^*†*^
Exp. 1′	AD	2 stages	Kendall's τ_*b*_	None	10		154	0.4824 (0.0319)^*†*^
Exp. 2′	AD	3 stages	Kendall's τ_*b*_	None	6		160	0.4298 (0.0574)^*†*^
fExp. 3′	Aβ	5 stages	Kendall's τ_*b*_	None	4.5		81	0.3638 (0.0590)^*†*^
Exp. 1-1	AD	2 stages	BCE	Laplacian	7	*e* ^−12^	360	0.8445 (0.0263)^*‡*^
Exp. 5-1	Aβ	Continuous	Pearson's *r*	Laplacian	8	*e* ^−16^	126	0.7019 (0.0527)^§^

* 5-fold CV performances are given as the mean and standard deviation.

† 5-fold CV performances were computed for Kendall's τ_*b*_.

‡ 5-fold CV performances were computed for accuracy.

§ 5-fold CV performances were computed for Pearson's *r*.

CV, Cross-validation; Exp., Experiment; AD, Alzheimer's disease; EOAD, Early-onset AD; LOAD, Late-onset AD; BCE, Binary cross-entropy.

### 3.1. Continuous disease-related alteration in BASIC scores

**Figure 2:**
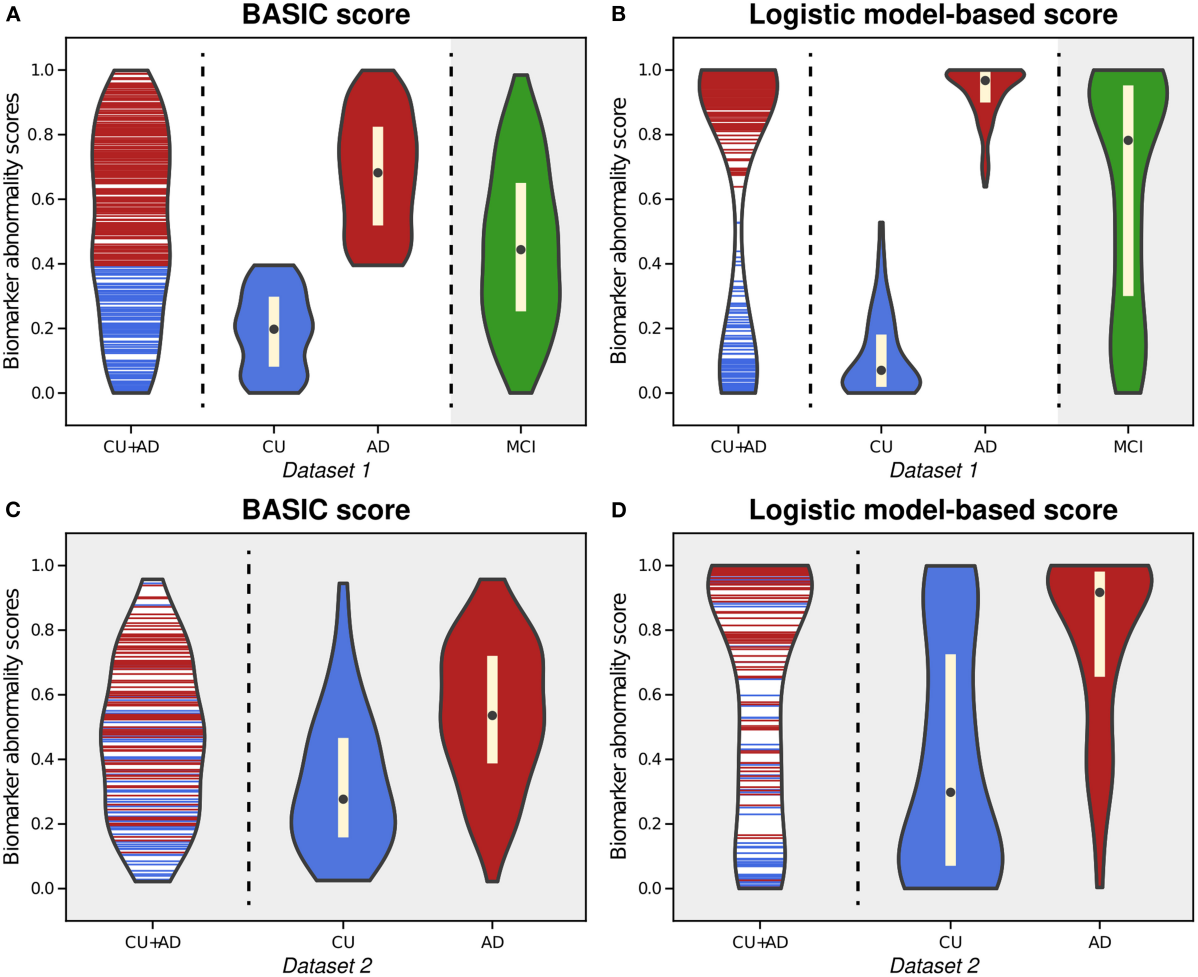
Violin plots illustrating the estimated distributions of BASIC scores **(A)** and logistic model-based scores **(B)** from whole-brain functional connectivity networks of cognitively unimpaired (CU) individuals and patients with Alzheimer's disease (AD) in Dataset 1. We also calculated the scores of patients with mild cognitive impairment (MCI) using both methods with parameter derived from CU and AD people (gray-shaded areas in **(A,B)**. For external validation, we calculated the BASIC scores **(C)** and logistic-model based scores **(D)** of CU and AD individuals in Dataset 2 from parameters obtained using CU and AD individuals in Dataset 1.

As an example of a neurodegenerative disorder, we first computed the BASIC scores associated with the presence or absence of AD for Dataset 1, (*Experiment 1*). By examining the distribution of estimated scores for all CU individuals and AD patients, we found that the likelihood of intermediate scores was comparable to that of extreme CU or AD scores (Figure 2A). This phenomenon differed from the biomarker abnormality scores computed using the logistic model (*Experiment 1-1*), demonstrating a higher likelihood of extreme CU and AD scores and a very low likelihood of the intermediate scores (Figure 2B).

Upon estimating the distributions for CU and AD individuals, the distribution of BASIC scores in each group was completely distinguished, as maximizing Kendall's τ_*b*_ with a dichotomous variable equivalent to maximizing the area under the receiver operating characteristic (AUROC) curve (Hanley and McNeil, 1982; Brossart et al., 2018). However, the score distributions of the CU and AD groups were differently shaped for each method. In contrast to the logistic model-based scores, we discovered that the BASIC scores were evenly distributed within each score range for each group.

When scores of MCI patients in Dataset 1 were calculated with the parameters of the summarizing function obtained for CU and AD individuals in Dataset 1, their BASIC scores had the highest likelihood in the mid-scores (gray-shaded area in Figure 2A). Patients with MCI were expected to be distributed between CU and AD. In contrast, a higher likelihood was observed in the score range close to AD in the logistic model-based scores (gray-shaded area in Figure 2B). A similar trend was observed when the scores were computed for Dataset 2 (Figures 2C,D). This demonstrates that BASIC scores reflect disease progression along the AD continuum more accurately than logistic model-based scores.

### 3.2. BASIC scores for multiple disease stages

**Figure 3:**
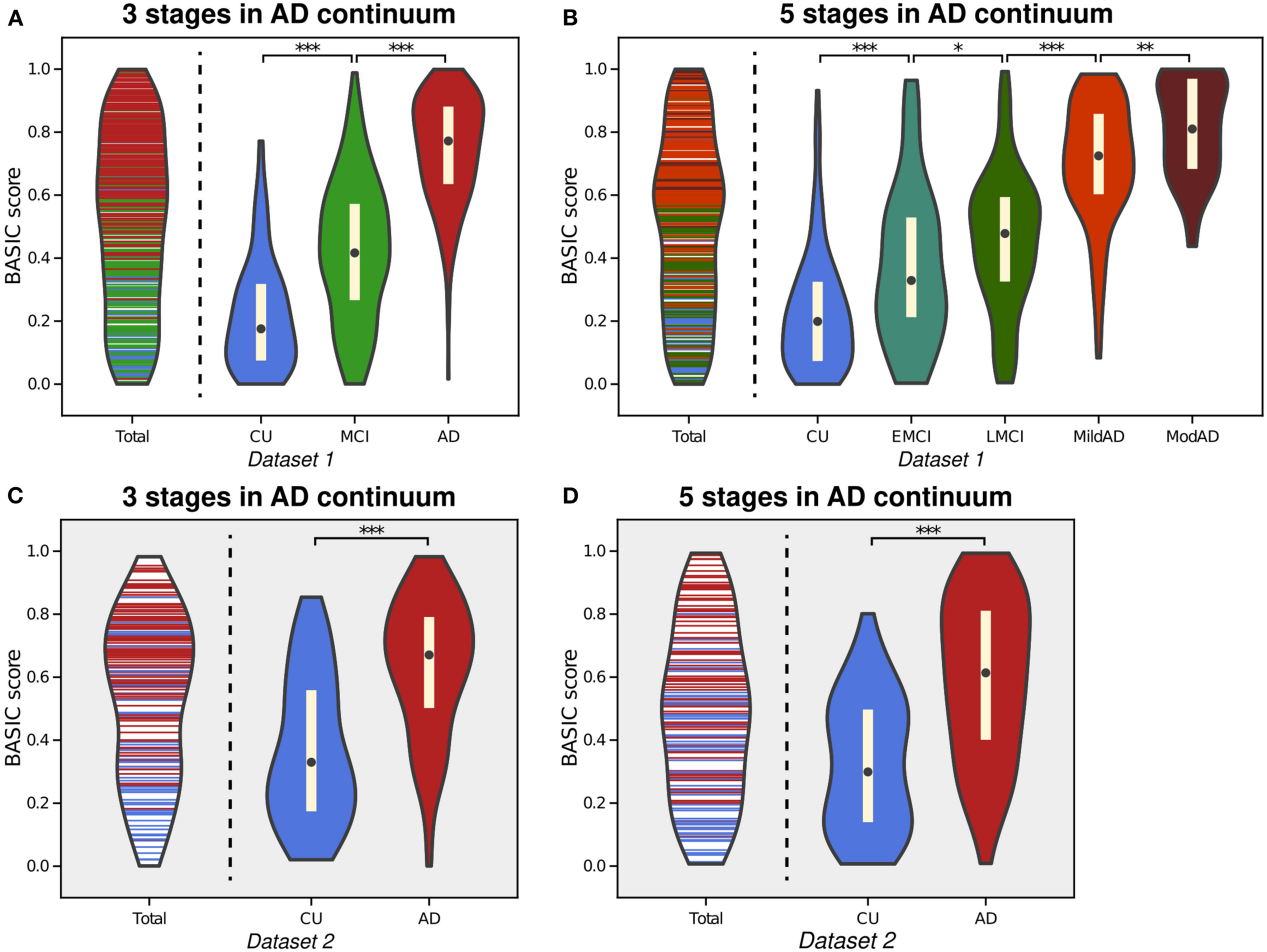
Violin plot depicting the estimated distributions of BASIC scores of individuals in Dataset 1 in a manner that maximizes the association with disease progression represented by either three stages **(A)** or five stages **(B)** in the Alzheimer's disease (AD) continuum. For external validation, we calculated the scores of people in Dataset 2 using parameters derived from three stages **(C)** and five stages **(D)**. *p < 0.05, **p < 0.01, and ***p < 0.001, with the false discovery rate correction for in each experiment.

AD is characterized by the gradual progression of cognitive deficits along a continuum, and its progression can be discretized into more than two consecutive stages. The proposed framework is applicable to this situation, producing BASIC scores that are associated with disease progression. Figure 3 depicts the estimated distributions when the stages on the AD continuum are three (CU, MCI, and AD; *Experiment 2)* and five (CU, EMCI, LMCI, MildAD, and ModAD; *Experiment 3*). In each instance, the distributions of scores for CU individuals and AD patients were more naturally at their boundaries than when scores were determined using only two groups. The MCI patients' scores exhibited a bell-shaped distribution, with the highest likelihood between the CU and AD scores. When using five groups, the median difference between LMCI and MildAD was larger than that between EMCI and LMCI or between MildAD and ModAD, indicating that BASIC scores can reflect monotonic nonlinear changes across disease stages.

**Figure 4:**
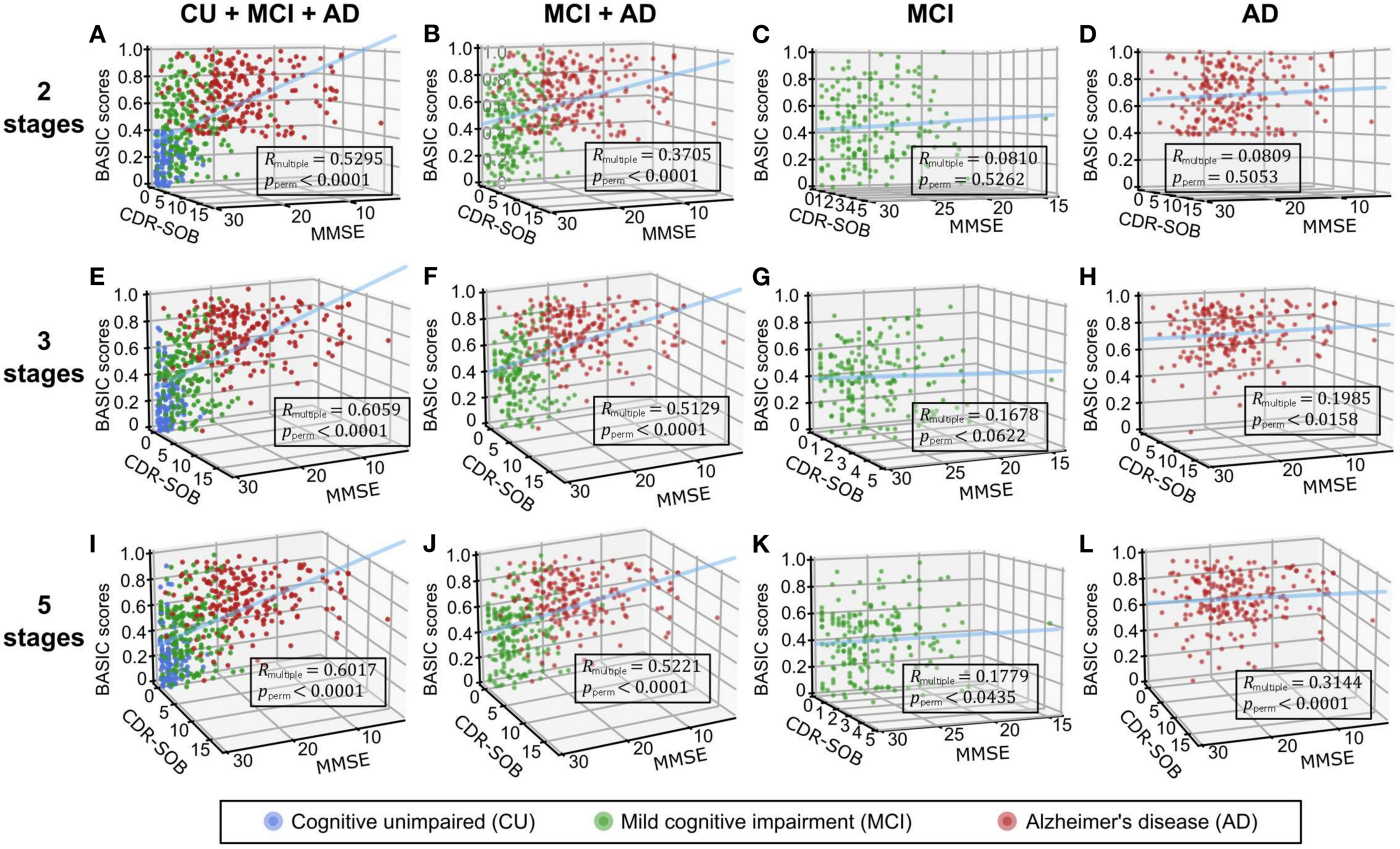
Three-dimensional regression plots with cognitive functioning scores, including the mini-mental state examination (MMSE) and clinical dementia rating scale-sum of boxes (CDR-SOB) as independent variables and BASIC scores as dependent variables. Participants in two **(A-D)**, three **(E-H)**, and five **(I-L)** stages of Alzheimer's disease (AD) were used to determine the BASIC scores. The coefficients of multiple correlations (*R*_*multiple*_) between BASIC scores and cognitive functioning scores were represented as *p*-values (*p*_*perm*_) using 10,000 permutations for each plot. CU, cognitively unimpaired; MCI, mild cognitive impairment.

We performed correlation analyses between the BASIC scores determined using different numbers of stages on the AD continuum and cognitive functioning scores directly related to AD (Figure 4). When BASIC scores were calculated using only two stages, there were significant correlations for all participants as well as only patients with dementia (including both MCI and AD patients). However, within each of the MCI and AD groups, no significant correlation was found. All associations were strengthened when disease stages were further subdivided, particularly within the MCI and AD groups, implying that the greater the number of disease stages utilized, the more truthful the biomarker score.

### 3.3. Edge importance in connectivity-based biomarkers

**Figure 5:**
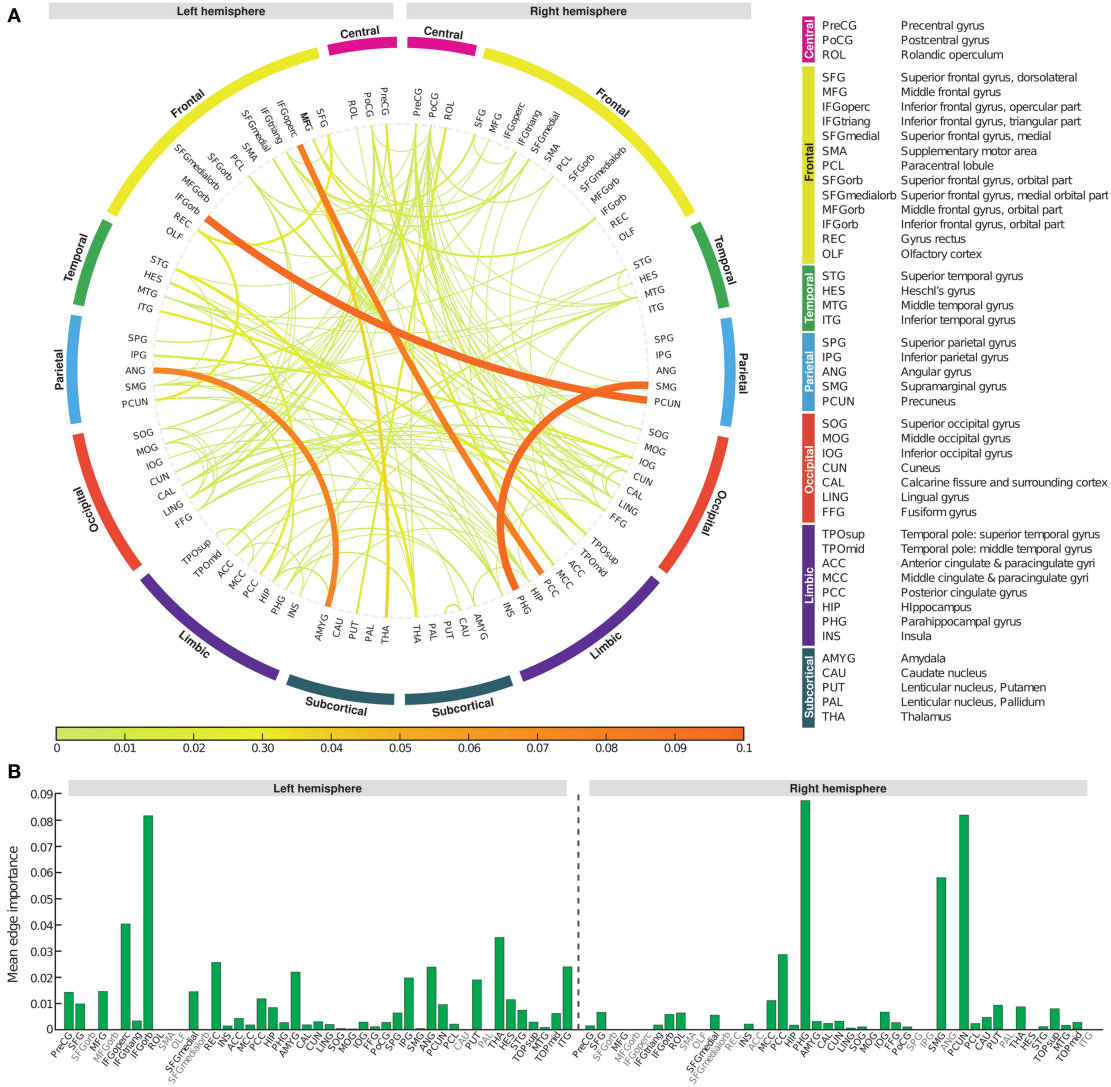
Edge importance profiles for connectivity-based biomarkers associated with Alzheimer's disease progression represented by the five stages. **(A)** A connectogram in which line thickness and color indicate the corresponding edge importance value. **(B)** The average value of edge importance for each brain region's incident edges.

To determine which edge had the greatest impact on biomarker scores, we defined *edge importance* as the square of the parameter values on edges, similar to how feature importance is calculated in linear regression (Grömping, 2015). As a representative example, the profile of edge importance in constructing BASIC scores associated with AD progression represented by the five stages is illustrated in Figure 5. Connections with high edge importance were incident to a set of regions, including the opercular/orbital parts of the left inferior frontal gyrus, left thalamus, right parahippocampal gyrus, right supramarginal gyrus, and right precuneus, where functional connectivity is altered as AD progresses (Grady et al., 2001; Wang et al., 2007; Zhou et al., 2013; Li et al., 2017).

### 3.4. Disease trajectory-specific BASIC scores

**Figure 6:**
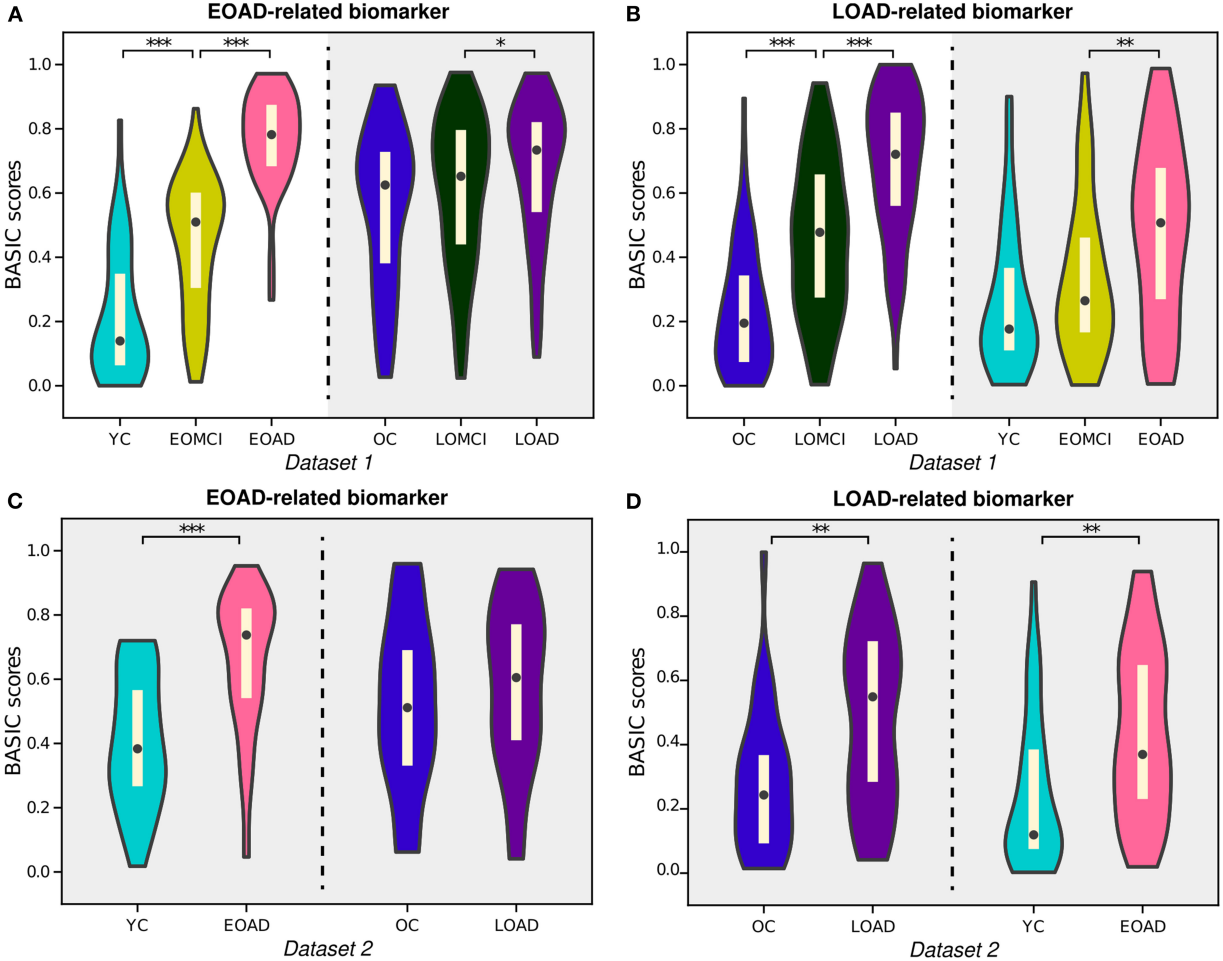
Violin plots depicting the estimated distributions of BASIC scores associated with the progression of early-onset Alzheimer's disease (EOAD) and late-onset Alzheimer's disease (LOAD). **(A)** EOAD-related biomarker scores were determined using three st ages in the EOAD trajectory using Dataset 1, including young cognitively unimpaired (YC) individuals and patients with early-onset mild cognitive impairment (EOMCI) and EOAD. The scores of the individuals in Dataset 1 were also calculated for the LOAD trajectory, which included old cognitively unimpaired (OC) individuals and patients with late-onset mild cognitive impairment (LOMCI) and LOAD. **(B)** LOAD-related biomarker scores were similarly determined for the individuals in Dataset 1. **(C)** EOAD-related biomarker scores of individuals in Dataset 2 were computed for the corresponding disease trajectories with parameters obtained from Dataset 1. **(D)** LOAD-related biomarker scores of individuals in Dataset 2 were computed for the corresponding disease trajectories with parameters obtained from Dataset 1. The *p*-values (*p*_*perm*_) were computed by permutation testing with 10,000 permutations. *p < 0.05, **p < 0.01, and ***p < 0.001, with the false discovery rate correction for in each experiment.

AD can be subdivided according to various criteria, one of which is the subtype divided by onset age: EOAD and LOAD. Previous research has shown that EOAD and LOAD can be distinguished by their distinct pathological and clinical characteristics, including patterns of cortical atrophy, white matter disruption, and cognitive impairment (Panegyres and Chen, 2013; Mendez, 2019; Lee et al., 2021b). In this study, our proposed framework provided BASIC scores for EOAD-related biomarkers based on EOAD trajectory (YC-EOMCI-EOAD) and LOAD-related biomarkers based on LOAD trajectory (OC-LOMCI-LOAD), respectively (*Experiment 4a* and *4b*). BASIC scores for the EOAD-related and LOAD-related biomarkers were sensitive to disease progressions of each subtype (left parts of Figures 6A,B).

Notably, individuals in the LOAD trajectory had relatively high BASIC scores for the EOAD-related biomarkers; in contrast, individuals in the EOAD trajectory had relatively low LOAD-related biomarker scores (gray-shaded areas in Figures 6A,B). Similar tendencies were observed for individuals in Dataset 2 when their BASIC scores were calculated using the parameters estimated from Dataset 1 (Figures 6C,D). This is in line with previous results that found that LOAD has a typical memory-disorder manifestation, whereas EOAD is characterized by deficits in the non-memory domain, such as visuoconstructional skills, executive function, and memory function (Joubert et al., 2016; Tellechea et al., 2018). In other words, the symptoms of EOAD are more extensive than those of LOAD, and features contributing to the EOAD-related biomarker score may also be present in LOAD, resulting in relatively high EOAD-related biomarker scores for individuals in the LOAD trajectory. Similarly, the LOAD-related biomarker score may be lower in EOAD because LOAD-related scores do not include all characteristics associated with EOAD. It is also worth noting that comparing the score distributions of stages on different disease trajectories, beyond comparing degrees of increases for the trajectories, may not be meaningful, because age or other demographic/clinical variables are not considered when constructing the score. These variables can distort the level of biomarker abnormality due to their association with disease manifestation; their effect can be controlled in further analyses that use the scores.

### 3.5. Construction of BASIC scores using continuous measures

**Figure 7:**
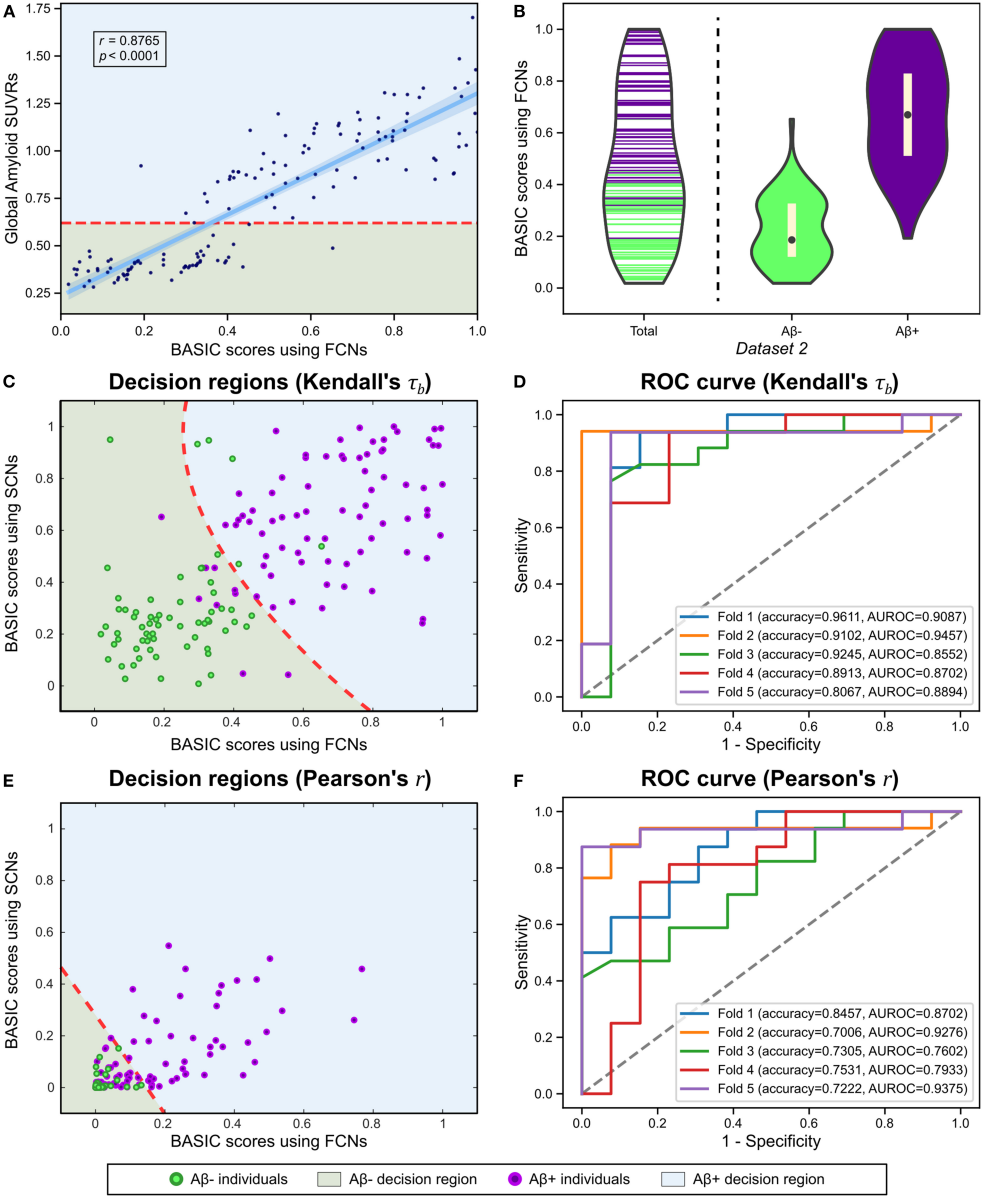
Using functional connectivity networks (FCNs), the BASIC scores were determined in a manner that maximizes Kendall's rank correlation coefficient (τ_*b*_) with the global amyloid standard uptake value ratio (SUVR) measured on a continuous scale. **(A)** Regression plot for the BASIC scores and the global amyloid SUVR. **(B)** Violin plots depicting the estimated distributions of the BASIC scores of individuals in Dataset 2 according to amyloid status (*i.e*., amyloid positive or negative). **(C)** Decision regions and boundary derived from the classification analysis using the BASIC scores using FCN and structural connectivity networks (SCNs), determined in an manner that maximizes Kendall's τ_*b*_. **(D)** The corresponding receiver operating characteristic (ROC) curves for five-fold cross-validation (CV). **(E)** Decision regions and boundary derived from the classification analysis using BASIC scores using FCNs and SCNs, determined in a manner that maximizes the Pearson's product-moment correlation coefficient (Pearson's *r*). **(F)** The corresponding ROC curves for five-fold CV.

There is a need to identify biomarkers associated with continuous pathological changes, which are sometimes the direct criteria for brain disorders. For instance, amyloid deposition is a necessary condition for diagnosing AD (Jack et al., 2018); consequently, determining whether an individual is amyloid-positive or amyloid-negative is essential for distinguishing AD from other neurodegenerative disorders, which can be determined by global retention values obtained from amyloid PET. We identified a brain connectivity-based biomarker and computed its score in relation to the global amyloid SUVR (*Experiment 5*). The BASIC scores were significantly correlated with the SUVR (Figure 7A). In addition, the distributions of scores in the amyloid-negative (Aβ-) and amyloid positive (Aβ+) groups were smoothly connected, with scores also present near the boundary between the two groups (Figure 7B).

We also performed a classification analysis between Aβ- and Aβ+ groups using BASIC scores derived from FCNs and structural connectivity networks (SCNs) from a previous study (Lee et al., 2021b). When classification was performed using BASIC scores obtained by maximizing Kendall's τ_*b*_, the two groups were distinguished with a five-fold CV accuracy of 0.8987 (Figures 7C,D). However, BASIC scores obtained by maximizing the canonical correlation coefficient, or equivalently, Pearson's *r* (*Experiment 5-1*) demonstrated poorer classification performance (5-fold CV accuracy of 0.7504), with a high rate of misclassification for the Aβ+ group (Figures 7E,F). This indicates that the association between the global amyloid SUVR and connectivity-based biomarkers cannot be explained solely by a linear relationship. This nonlinear relationship can be detected more effectively using Kendall's τ_*b*_ than Pearson's *r*.

## 4. Discussion

Given the importance of studying brain connectivity in neurodegenerative disorder research, it is critical to identify connectivity-based biomarkers associated with disease progression. Moreover, because multiple pathologies are involved in explaining or predicting neurodegenerative disorders, summarizing these biomarkers into a single numerical value is beneficial. Previous studies have used various multivariate statistical techniques to either extract biomarkers from brain connectivity or transform them into a single score (Zalesky et al., 2010; Shen et al., 2017). We established a framework that unifies these two respective steps in a manner that maximizes the nonlinear monotonic relationship to disease stages represented either continuously or discretely, providing BASIC scores that are sensitive to disease progression as well as specific to a particular disease trajectory. In addition, the BASIC scores successfully captured continuity in brain changes along with disease progression, with superior generalizability.

It is natural that biomarker scores change continuously as the disease advances, given that the associated pathological phenomenon also changes continuously as the disease progresses. In this context, individuals with moderate conditions cannot be ignored in comparison to patients with severe conditions or healthy individuals, which should be reflected in the biomarker scores. From this perspective, the BASIC scores were not only distributed overlapping of each disease stage but were also approximately evenly distributed between the upper and lower bounds of the scores, which was not observed in the responses of the logistic models. This could be made possible by freeing the binary prediction model's assumption that the outcome follows the Bernoulli distribution and instead maximizing the rank correlation. In addition, the BASIC score, computed by the weighted sum of connection strengths and transformation by the normal CDF, approximately followed a uniform distribution according to the central limit theorem; thus, the proportion of people with intermediate scores was greater than those with intermediate binary prediction model responses.

Our framework was designed to be generalizable in providing connectivity-based biomarker abnormality scores. This is mainly accomplished by employing a regularization procedure, in which additional prior information is introduced to obtain parameters and subsequently construct scores. First, the structural attributes inherent to brain connectivity were considered in the form of Laplacian regularization, which produces a smoothing effect on adjacent edges. If one of the adjacent edges has extreme connection strengths, its parameter will have a value similar to that of the adjacent edges. Hence, their connection strengths were affected at a similar level when constructing the score. Comparing five-fold CV performances with and without Laplacian regularization in our framework's objective function, we empirically validated that Laplacian regularization produced superior generalized performance in reflecting the stage of the disease (*Experiment 1* vs. *1*′, *Experiment 2* vs. *2*′, and *Experiment 3* vs. *3*′ in Table 3). Controlling the extent of a connectivity-based biomarker and choosing the most parsimonious one based on the 1SE rule also reduced the influence of unseen connectivity by decreasing the number of variables used in the score computation. In addition, the framework measured the association with the reference variable using Kendall's rank correlation, which may affect generalizability by mitigating the effect of connection strengths caused by measurement errors. Finally, the framework selects optimal hyperparameter values based on CV, which prevents data overfitting (Hastie et al., 2009; Shen et al., 2017).

Identifying reliable biomarkers, regardless of their abnormality score, is the focuses of this framework. It is also possible, for instance, to estimate parameters by maximizing the association through explicit regularization while controlling the sparsity for the entire edge set. However, the collection of selected edges is inappropriate for use as a biomarker because ae sparsity-controlling procedure may not produce a unique solution. The framework proposed in this study identified a biomarker with a connectivity-specific attribute by leveraging a NBS, which identifies the effect of interest with high statistical power (Zalesky et al., 2010). Moreover, given that brain connectivity should be viewed as a complex system that regulates neural information flow, the biomarker extracted by the multivariate technique combined with mass-univariate hypothesis testing can explain network-topological alterations, as in previous research (Myung et al., 2016; Kim et al., 2017, 2019). This is why our framework is not designed as a single method but instead consists of two separable techniques.

There are several limitations to this study. We used the continuum of clinical manifestation of AD as an approximation of the AD continuum when considering continuous brain alterations for demonstrating the continuity of BASIC scores. Despite this, it is believed that demonstrating the capability of our framework is not a concern because it maximizes the association to any disease stage represented as ordinal scales. In addition, although BASIC scores capture nonlinear monotonic relationships through Kendall's τ_*b*_, it is limited in its ability to accurately reflect alterations in brain connectivity as the disease progresses. Suppose that multiple connected components are extracted in the identification step, showing distinct dynamic patterns of changes. In this case, linear summarization is insufficient to reflect the complex nonlinear relationship with the behavior of the biomarker. As AD progresses, the default mode network (DMN) and salient network exhibit an inverse relationship with functional connectivity (Brier et al., 2012; Zhou and Seeley, 2014). Furthermore, functional connectivity in DMN regions has been reported to have a quadratic relationship with disease progression (Schultz et al., 2017); however, this tendency cannot be measured using Kendall's τ_*b*_. In the future, we intend to use graph-based deep-learning models to capture and summarize these complex nonlinear relationships (Kipf and Welling, 2017; Xu et al., 2018).

In conclusion, our unified framework provides an explainable connectivity-based biomarker and continuity-reflecting BASIC scores that are sensitive and specific to disease progression. The selection of NBS statistics during the identification step was flexible within the framework. In addition, the framework is not restricted by the type of specific association measure, thresholding criterion, or summarizing function used in the summarization step. The framework is applicable to other network-based neurodegenerative disorders, such as Parkinson's disease, progressive supranuclear palsy, and amyotrophic lateral sclerosis, where brain connectivity is disrupted as the diseases progress (Brown et al., 2017; Brundin and Melki, 2017; Romano et al., 2022), although this paper focuses on AD-related brain connectivity. With this flexibility, our framework establishes a milestone for analyzing complex brain connectivity networks to explain or predict neurodegenerative disorders by presenting indicators of the degree to which brain networks collapse or by utilizing them as one of the features for predictive models.

## Data availability statement

The raw data and/or codes supporting the conclusions of this article will be made available by the authors, without undue reservation.

## Ethics statement

The studies involving human participants were reviewed and approved by the Institutional Review Boards (IRB) of either Samsung Medical Center or Gachon University Gil Medical Center. The patients/participants provided their written informed consent to participate in this study.

## Author contributions

J-KS had full access to all the data in the study and take responsibility for the integrity of the data and the accuracy of the data analysis. J-KS and S-WK planned the study design, concept, interpreted the results, and wrote the original manuscript. HK, YN, SS, and DN collected image data. J-KS, S-WK, and Y-HS preprocessed image data and performed image-based analyses. All authors reviewed and edited the manuscript.

## Funding

This research was supported by the National Institute of Health research project (2022-ER1003-00), a grant from the Korea Healthcare Technology R&D Project through the Korea Health Industry Development Institute (KHIDI), and also funded by the Ministry of Health and Welfare of the Republic of Korea (Grant No HI14C1135) and a National Research Foundation of Korea (NRF) Grant funded by the Korean Government (MSIT) (No. 2018M3C7A1056889).

## Conflict of interest

The authors declare that the research was conducted in the absence of any commercial or financial relationships that could be construed as a potential conflict of interest.

## Publisher's note

All claims expressed in this article are solely those of the authors and do not necessarily represent those of their affiliated organizations, or those of the publisher, the editors and the reviewers. Any product that may be evaluated in this article, or claim that may be made by its manufacturer, is not guaranteed or endorsed by the publisher.
